# Psychosocial and Physical Activity Outcomes Among Group Lifestyle Balance Program Participants With Arthritis

**DOI:** 10.1093/geroni/igab046.1765

**Published:** 2021-12-17

**Authors:** Cheryl Der Ananian, Ferdinand Delgado, Taylor Hudzinski

**Affiliations:** 1 Arizona State University, Phoenix, Arizona, United States; 2 Arizona State University, Arizona State University, Arizona, United States

## Abstract

Background: Weight loss and physical activity (PA) are recommended for arthritis management. The Group Lifestyle Balance (GLB) Program(TM) is an evidence-based, lifestyle change program for weight loss in individuals with prediabetes, but hasn’t been evaluated in people with arthritis. Purpose: The purpose of this study was to evaluate the effectiveness of an adapted version of the GLB program on PA and psychosocial outcomes related to weight loss among overweight (Body Mass Index >27) individuals with arthritis. Methods: A single-group, quasi-experimental design was used to examine the effects of the adapted GLB program on measures of PA and psychosocial outcomes. All participants (N=15) received the GLB program and completed the following surveys: CHAMPS PA, Self-Efficacy for PA (SE), Social Support for PA (SS), Weight Loss Efficacy (WEL) and Barriers to Healthy Eating (BHE) at baseline, 12-weeks, 6 months, and 12 months. Repeated measures ANOVA and the Friedman Test were used to examine changes over time. Results: Participants (aged 53-79 years) were primarily female (82%), white (94%), and college educated (94%). Significant improvements were found in BHE subsections of self-control and motivation (p=0.002), daily mechanics (p=0.042), and WEL subsections of availability (p=0.049), social pressure (p=0.010), physical discomfort (p=0.011), and positive activities (p=0.007). Weekly caloric expenditure (p=0.004), metabolic equivalent minutes (p=0.022) for all activities, and moderate-intensity activities (p=0.019) also showed significant improvements. However, most improvements were seen in the short-term. Conclusions: The GLB program should be further evaluated for its effectiveness in people with arthritis.

